# Mandibular Osteomyelitis and Acute Soft Head Syndrome in an Adolescent With Sickle Cell Disease

**DOI:** 10.14740/jmc5366

**Published:** 2026-07-28

**Authors:** Abdulelah Abdulrahman Almugahwi, Rehab Yusuf Al-Ansari

**Affiliations:** a Internal Medicine Department, Hematology Unit, King Fahd Military Medical Complex, Dhahran, Saudi Arabia; b Department of Internal Medicine, College of Medicine, Imam Abdulrahman Bin Faisal University, Khobar, Saudi Arabia

**Keywords:** Sickle cell disease, Acute soft head syndrome, Mandibular osteomyelitis, Pain crises

## Abstract

Sickle cell disease (SCD) is a type of inherited disorder of hemoglobin characterized by recurring vaso-occlusive pain crises that affect many organ systems. Although it is uncommon, involvement of the orofacial region, such as the mandible area and jaw, can lead to a mandibular osteomyelitis, which can cause serious morbidity and diagnostic uncertainty. We describe a 17-year-old male Saudi patient with SCD who initially complained of bodily ache before developing fever, facial swelling, frontoparietal head swelling, and elevated inflammatory markers. The image resembled an odontogenic abscess along with frontoparietal head collection. However, the patient’s clinical history, painful crises, elevated inflammatory markers, and high Hb S% along with further radiological study were all compatible with a mandibular osteomyelitis along with acute soft head syndrome. Vigorous supportive care with exchange transfusions to lower Hb S%, antibiotic support, and multidisciplinary consultation was all part of the management. The patient was steadily getting better, and after 1 month from starting management, all of the facial and head swelling had completely disappeared. In conclusion, this case highlights the presentation of two uncommon SCD manifestations: mandibular osteomyelitis and acute soft head syndrome in young SCD patient. Additionally, it demonstrated how crucial it is to keep mandibular crisis (osteomyelitis) as a differential diagnosis for orofacial pain in SCD patients and how lowering Hb S% is important for treating such instances.

## Introduction

Sickle cell disease (SCD) is an inherited hemoglobinopathy and an autosomal recessive disease that is characterized by chronic hemolytic anemia and recurrent vaso-occlusive crises (VOCs) and pain crisis. While VOCs usually affect long bones, chest, and abdomen, orofacial involvement, particularly mandibular involvement, presents less commonly yet can result in significant morbidity [1, 2]. Severe pain, swelling, and neurological symptoms can be the manifestations of mandibular crisis that may mimic odontogenic infection, leading to diagnostic and therapeutic challenges [3, 4]. Osteomyelitis of the jaw or mandibular bone is an infrequent presentation of SCD, especially in young age patients [5]. On the other hand, soft head syndrome (subgaleal hematoma) is another uncommon presentation of SCD crisis and the data in the literature are limited to some scattered worldwide case reports with no guidelines on how to manage this situation [6, 7]. Therefore, we report a case of a male Saudi adolescent with SCD who experienced a mandibular crisis/osteomyelitis along with acute soft head syndrome (ASHS), highlighting the importance of early diagnosis and multidisciplinary management in this patient population.

## Case Report

This is a 17-year-old male Saudi patient, a known case of SCD since child hood. He continued to receive 5 mg of folic acid orally once daily and 500 mg of hydroxyurea twice daily, with reported non-compliance to his medication. He arrived at the emergency room complaining of 3 days of widespread body ache, consistent with his usual sickling pain, that was not responding to oral analgesics (paracetamol and ibuprofen). His past surgical history was remarkable for laparoscopic cholecystectomy 5 months prior to the current presentation. He had occasional admissions and a history of multiple blood exchange transfusions for acute chest syndrome. He also had a history of recurrent ASHS that relived with exchange transfusion, as well as a previous history of pulmonary embolism (PE) for which he was on 5 mg of apixaban twice daily. During the current presentation, he denied chest pain, shortness of breath, palpitations, abdominal pain, nausea, vomiting, change in bowel habits, weakness, loss of consciousness, seizures, vision changes, or urinary/respiratory symptoms.

Upon admission, physical examination revealed normal vital signs with a temperature of 36.6 °C. The patient appeared to be in severe pain, yet he was not pale or cyanosed. He was fully oriented with no neurological deficit. The head and neck examination revealed normal findings with considerable edema and swelling. An inspection of the abdomen revealed a scar from a prior laparoscopic treatment. Otherwise, there were no notable findings on the respiratory, cardiac, or genitourinary examinations.

Initial laboratory investigation showed hemoglobin (Hb) of 9.5 g/dL, Hb S of 77.3%, and lactate dehydrogenase (LDH) of 721 U/L; further investigation is shown in Table 1. Baseline chest X-ray was within normal limits and electrocardiogram showed normal sinus rhythm.

**Table 1:** Laboratory Results During Hospitalization and at the Time of Outpatient Follow-Up

Test name	D1	D2	D3	D4	D5	D6	D7	D8	D9	D10	D11	D12	D13	D14	D28 (OPD follow-up)
Hematology (reference range)															
WBC (4–11 × 10^3^/µL)	8.1	15.9	12.8	8.48	6.71	7.06	7.05	7.0	6.9	6.8	6.7	6.6	6.5	6.4	5.61
Hb (13–18 g/dL)	9.5	8.46	7.82	8.68	9.60	10.1	9.80	9.5	9.2	8.90	8.60	8.30	8.00	7.80	8.4
Hct (42–52%)	28.4	25.3	24.5	27.0	29.3	31.0	30.0	29	28	27.0	26.0	25.0	24.0	23.0	31
Plt (140–450 × 10^3^/µL)	497	236	231	242	221	248	245	240	235	230	225	220	215	210	232
HPLC															
Hb S (%)	77.3			55.7		32.3	29								37%
Chemistry (reference range)															
ALP (40–150 U/L)	208	579	565	350	251	234	229	225	220	215	210	205	200	195	145
ALT (7–40 U/L)	140	125	92	52	39	31	28	25	23	21	19	17	15	13	12
AST (5–34 U/L)	98	184	119	48	30	30	29	27	25	23	21	19	17	15	7
Total bilirubin (5–21 µmol/L)	52.3	91	65	56.7	42.7	53.2	37.7	35.6	33.4	31.2	29	26.8	24.6	22.4	11
CRP (Neg ≤ 5 mg/L)	26	177.9		382.4	356.4		358.9	345.6	330.2	315.8	300.4	285	270.6	250	4
LDH (125–220 U/L)	721	-	-	2,505	1,658	1,520	1,433	1,356	1,300	1,250	1,200	1,150	1,100	1,050	238

ALP: alkaline phosphatase; ALT: alanine aminotransferase; AST: aspartate aminotransferase; CRP: C-reactive protein; D: day; Hb: hemoglobin; Hb S: hemoglobin S; Hct: hematocrit; HPLC: high-performance liquid chromatography; LDH: lactate dehydrogenase; OPD: outpatient department; Plt: platelet count; WBC: white blood cell.

The patient was admitted to the general ward bed with the admission diagnosis of SCD with VOC. Intravenous (IV) fluid of dextrose 5% normal saline at 120 mL/h was initiated along with pain killer medication in form of morphine 5 mg subcutaneous every 4 h as needed for sever pain, paracetamol 1 g IV four times daily, and ibuprofen 400 mg (1 tablet) orally three times daily.

### Hospital course

#### Day 1

The patient experienced severe body pain, with a fever of 37.7 °C. chest X-ray requested which showed no acute lung shadowing and septic workup (two sets of blood cultures and urine culture) were obtained. He was started empirically on ceftriaxone 2 g IV once daily and azithromycin 500 mg IV once daily due to his previous history of recurrent acute chest syndrome.

#### Day 2

The patient was still spiking fever up to 38.5 °C, with new tender, firm, right mandibular swelling (oval shape 3 × 5 cm) and new non-tender, fluctuated, right frontoparietal head swelling (round 5 × 6 cm). Laboratory results showed elevated inflammatory and hemolysis markers (Table 1). Dental consultation was ordered due to suspicion of dental swelling and abscess. Bedside dental X-ray showed no active dental infection. Despite this, the infectious disease (ID) team reported possible dental swelling, most likely abscess. Due to the extremely high fever and inflammatory markers, and the suspicion of dental involvement, a dentist consultation was ordered where the expertise excluded dental involvement although the patient complained of pain on the right side of mandible/jaw. ID team was consulted and the antibiotic was intensified to tazobactam.

#### Day 3

A plan for manual blood exchange transfusion in the intensive care unit (ICU) was made with manual exchange transfusion protocol as per hospital policy to reduce Hb S%, which was reduced down to 29%. Furthermore, we continued the management lines that were initiated previously, such as administering balanced parenteral hydration and appropriate analgesia, in addition to follow-up with ID and obeying their decision regarding the antibiotics and monitoring the cultures.

#### Day 4

Ongoing hemolysis resulted in a decline in Hb level to 5.5 g/dL, necessitating a one-unit top-up blood transfusion.

#### Day 5

Laboratory results showed partial recovery in some of the hematological indices but ongoing signs of hemolysis and inflammation (Table 1). The facial and head swelling was gradually improving. During this time, all blood and urine cultures came to be negative for any growth. Due to the increase in procalcitonin up to 4.3, the antibiotics were further intensified to vancomycin and meropenem.

#### Day 6

**Figure 1:**
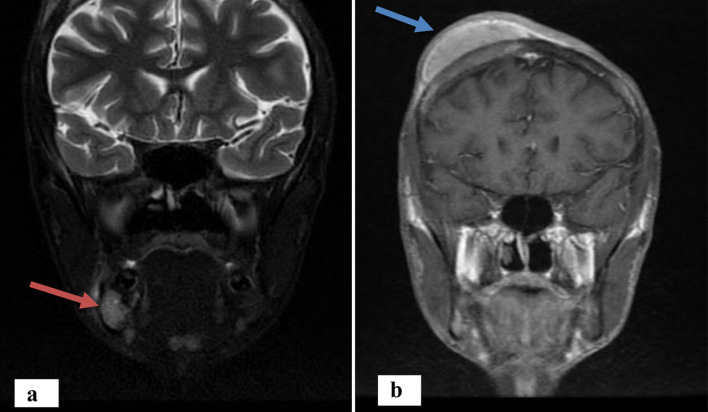
Brain and face MRI of the patient. (a) Osteomyelitis of the right mandible (red arrow). (b) Diffuse scalp swelling, frontoparietal regions, consistent with soft head syndrome (blue arrow). MRI: magnetic resonance imaging.

The patient was vitally stable and afebrile, without any fever spikes in the last 24 h. Facial and dental swelling improvement was progressing. Swelling over the scalp continued but without any inflammation, as was anticipated for soft head syndrome. Furthermore, the magnetic resonance imaging (MRI) of the brain and face with contrast was performed which showed normal brain parenchyma with no evidence of acute infarction, hemorrhage, or mass lesion. However, the facial MRI revealed diffuse thickening and enhancement of the soft tissues overlying the right mandible, with associated marrow edema. No discrete fluid collection or abscess was identified. These findings were consistent with osteomyelitis of the mandible, likely secondary to a VOC. Additionally, diffuse scalp swelling, particularly in the frontal and parietal regions, was noted, consistent with soft head syndrome (Fig. 1). Neurosurgery colleagues were also consulted, but no surgical intervention was warranted.

#### Day 7 to day 14

The patient remained afebrile and vitally stable. The facial and scalp swelling continued to resolve. The laboratory markers of inflammation and hemolysis continued to decrease. The patient completed the course of IV antibiotics and was switched to oral. He was discharged in a stable condition with an arrangement for follow-up in 2 weeks’ time in the hematology clinic.

#### Day 28 (outpatient department follow-up)

The patient was totally free of pain and afebrile, with complete resolution of the right mandibular and frontoparietal swellings.

## Discussion

The case illustrates the diagnostic challenge of a mandibular VOC in a young male Saudi patient with SCD that initially presented with probable symptoms and signs of a dental abscess, especially with rising inflammatory markers with this presentation. The definitive diagnosis was made by careful observation of the clinical course, laboratory results, and MRI. Although orofacial pain and swelling have been reported as presentations of VOC in SCD, they remain uncommon [1, 2]. A preliminary suspicion of an odontogenic infection was reasonable given the symptoms at presentation. However, a paucity of findings on dental imaging, in conjunction with the patient’s known SCD and markers of hemolytic activity—elevated LDH, bilirubin, and reticulocyte count—favored a diagnosis of mandibular VOC. Markedly elevated inflammatory markers, C-reactive protein (CRP) and procalcitonin, also leaned towards this diagnosis as infection is usually a potent precipitant of VOCs [8].

MRI revealed diffuse marrow edema and soft tissue enhancement of the mandible without a discrete abscess, consistent with bone infarction secondary to vaso-occlusion [9]. Bone infarction must be differentiated from pyogenic abscess, since these entities have very different therapeutic approaches; while abscesses typically require surgical drainage, infarctions are treated medically. The concomitant development of an acute episode of “soft head syndrome,” another manifestation of bone infarction in SCD, was strong evidence for the diagnosis of a systemic VOC.

Additionally, vaso-occlusive events with limited vascular supply can affect the maxillofacial region, leading to osteomyelitis of the jaws/mandible. Osteomyelitis is typically found in long bones, but can occur elsewhere. Laboratory tests cannot distinguish osteomyelitis from VOC with certainty. Moreover, high white blood cell and inflammatory markers can be present in both and differentiation is difficult without a positive bacterial culture. However, a negative culture does not exclude the diagnosis of osteomyelitis [10].

Osteomyelitis in SCD patients typically requires 4–6 weeks of treatment with antibiotics, which can be administered intravenously for at least 2 weeks before switching to oral antibiotics for the remainder of the course of treatment if the patient responds to initial treatment [1, 10, 11]. Antibiotic selection is based on the results of the culture; however, information about antibiotic selection in SCD patients with osteomyelitis is limited, particularly in negative cultures as the one in our instance [12].

However, in SCD patients, ASHS, also known as subgaleal hematoma, is characterized by sculp swelling with or without tenderness as a result of severe infarction and VOC, culminating in hemorrhage under the galeal neuronal tissue. MRI is necessary to identify this illness. Unfortunately, there is a deficit of information regarding ASHS, and what is known emphasizes conservative care over surgical intervention. Inaccurate diagnosis can result in needless surgical procedures [13].

Management herein was multidisciplinary and in line with current best practice. Aggressive analgesia and hydration remain the mainstay of VOC management. Exchange transfusion was dramatic in its effect by rapidly reducing the percentage of sickle Hb, thereby improving microvascular perfusion and interrupting the cycle of vaso-occlusion [14]. Broad-spectrum antibiotics were indicated by markedly elevated inflammatory markers and the inability to exclude a superimposed infection with certainty, a recognized and potentially life-threatening complication in SCD [15]. Antibiotic therapy was appropriately adjusted according to clinical improvement and ID consultation.

The case has particular significance in Saudi Arabia, where SCD is prevalent [16]. The clinical and radiological appearance is in line with ASHS and orofacial manifestations in SCD that have been previously documented in the local population [17]. It underscores the fact that clinicians working in areas of high prevalence need to have a high index of suspicion for mandibular VOC when orofacial pain and swelling occur, even in the absence of apparent dental pathology. Timely, accurate diagnosis and a coordinated multidisciplinary approach among hematology, ID, and dental and neurosurgery specialists is critical to both maximizing patient outcomes and preventing long-term sequelae such as chronic osteomyelitis or nerve impairment.

In fact, Saudi Arabia has a high burden of SCD, hence early diagnosis is crucial. Saudi Arabia presently relies on premarital and targeted high-risk screening for SCD, despite universal newborn screening being normal practice in nations like the United States and the United Kingdom. This disparity emphasizes the necessity of more extensive neonatal screening initiatives in order to guarantee prompt diagnosis and treatment [18–22].

Furthermore, in contrast to the United States and the United Kingdom, Saudi Arabia does not publicly designate Centers of Excellence (CE) for SCD. However, due to their large patient volumes, specialized hematology services, interdisciplinary teams, and expertise handling complex SCD complications, including uncommon manifestations like jaw osteomyelitis and ASHS, a number of hospitals serve as conventional national referral hubs. Thus, the demand of EC in every Saudi Arabian province could aid in future plans to improve the quality of care for SCD patients.

### Conclusion

A rare but potentially fatal SCD consequence, mandibular crisis in the form of osteomyelitis can mimic common orofacial and dental conditions. However, another uncommon manifestation of SCD patients is ASHS. This case highlights the significance of a high index of suspicion, a comprehensive diagnostic workup that includes modern imaging, and a multidisciplinary approach to therapy. It also demonstrates the therapeutic and diagnostic challenge of this disorder. Health care providers must be aware of mandibular VOC in areas where SCD is prevalent, like Saudi Arabia, in order to detect and treat patients early on, which can lower morbidity and enhance their quality of life. Additionally, there are no effective recommendations for treating rare illnesses like the one we dealt. In this instance, we found that lowering Hb S% and providing antibiotic treatment improved outcomes for SCD patients with mandibular osteomyelitis and/or ASHS. National or international standards are required to standardize the management of orofacial problems in SCD.

## Data Availability

The authors declare that data supporting the findings of this study are available within the article.
